# The management impact of ^68^gallium-tris(hydroxypyridinone) prostate-specific membrane antigen (^68^Ga-THP-PSMA) PET-CT imaging for high-risk and biochemically recurrent prostate cancer

**DOI:** 10.1007/s00259-019-04643-7

**Published:** 2019-12-23

**Authors:** Meghana Kulkarni, Simon Hughes, Andrew Mallia, Victoria Gibson, Jennifer Young, Ajay Aggarwal, Stephen Morris, Ben Challacombe, Rick Popert, Christian Brown, Paul Cathcart, Prokar Dasgupta, Victoria S. Warbey, Gary J. R. Cook

**Affiliations:** 1 Urology Centre, Guy’s & St Thomas’ NHS Trust, London, SE1 7EH UK; 2 Cancer Imaging Department, School of Biomedical Engineering and Imaging Sciences, King’s College London, London, UK; 3 Department of Oncology, Guy’s and St Thomas’ Hospitals NHS Trust, London, UK; 4 Department of Nuclear Medicine, Guy’s and St Thomas’ Hospitals NHS Trust, London, UK; 5 Department of Chemistry and Biology, School of Biomedical Engineering and Imaging Sciences, King’s College London, London, UK

**Keywords:** Prostate-specific membrane antigen, Prostate cancer, PSMA-PET-CT, Management impact, ^68^Ga-THP-PSMA

## Abstract

**Purpose:**

To determine the impact on clinical management of patients with high-risk (HR) prostate cancer at diagnosis and patients with biochemical recurrence (BCR) using a new kit form of ^68^Ga-prostate-specific membrane antigen (PSMA), namely tris(hydroxypyridinone) (THP)-PSMA, with positron emission tomography-computed tomography (PET-CT).

**Methods:**

One hundred eighteen consecutive patients (50 HR, 68 BCR) had management plans documented at a multidisciplinary meeting before ^68^Ga-THP-PSMA PET-CT. Patients underwent PET-CT scans 60-min post-injection of ^68^Ga-THP-PSMA (mean 159 ± 21.2 MBq). Post-scan management plans, Gleason score, prostate-specific antigen (PSA) and PSA doubling time (PSAdt) were recorded.

**Results:**

HR group: 12/50 (24%) patients had management changed (9 inter-modality, 3 intra-modality). Patients with PSA < 20 μg/L had more frequent management changes (9/26, 34.6%) compared with PSA > 20 μg/L (3/24, 12.5%). Gleason scores > 8 were associated with detection of more nodal (4/16, 25% vs 5/31, 16.1%) and bone (2/16, 12.5% vs 2/31, 6.5%) metastases. BCR group: Clinical management changed in 23/68 (34%) patients (17 inter-modality, 6 intra-modality). Forty out of 68 (59%) scans were positive. Positivity rate increased with PSA level (PSA < 0.5 μg/L, 0%; PSA 0.5–1.0 μg/L, 35%; PSA 1.0–5.0 μg/L, 69%; PSA 5.0–10.0 μg/L, 91%), PSAdt of < 6 months (56% vs 45.7%) and Gleason score > 8 (78.9% vs 51.2%).

**Conclusions:**

^68^Ga-THP-PSMA PET-CT influences clinical management in significant numbers of patient with HR prostate cancer pre-radical treatment and is associated with PSA. Management change also occurs in patients with BCR and is associated with PSA and Gleason score, despite lower scan positivity rates at low PSA levels < 0.5 μg/L.

## Introduction

Prostate cancer is the most commonly diagnosed malignancy in men and is a leading cause of cancer-related death [1, 2]. Accurate staging of prostate cancer before radical treatment and for the detection of recurrence is vital for directing treatment and predicting prognosis [3]. This has conventionally been dependent upon digital rectal examination, prostate-specific antigen (PSA) testing and prostate biopsy, complemented with imaging, including multiparametric MRI (mpMRI), computed tomography (CT) and bone scintigraphy [4].

More recently, positron emission tomography-computed tomography (PET-CT), with tracers such as ^18^F-choline, has increasingly played a role in the management of these patients [5, 6]. However, sensitivity is only moderate for detecting nodal metastases [5] or in those with low PSA levels at biochemical recurrence (BCR) [7]. This has led to the investigation of more prostate-specific tracers with greater diagnostic accuracy.

Prostate-specific membrane antigen (PSMA) is a type II transmembrane glycoprotein with significantly increased expression in prostate cancer cells compared with benign tissue [8]. It has emerged as a potential disease biomarker for imaging and targeted treatment. Several enzymes with structural and functional homology to PSMA have been identified leading to the possibility of exploiting these small molecules for the imaging and treatment of prostate cancer via PSMA-targeting [9].

The clinical breakthrough of PET imaging with PSMA ligands was achieved with ^68^Ga-HBED-CC-PSMA, also referred to as ^68^Ga-PSMA-11 [10, 11]. This compound has a strong binding affinity for PSMA and highly efficient internalisation into the prostate cancer cell. Since 2011, the use of PSMA PET ligands, predominantly ^68^Ga-HBED-CC-PSMA, has gained rapid acceptance with excellent diagnostic performance in BCR, with an ability to detect lesions at low PSA levels, but also in primary staging [9, 12, 13].

A novel cold-kit formulation, ^68^Ga-tris (hydroxypyridinone)-PSMA (^68^Ga-THP-PSMA), has been introduced that allows more rapid radiolabelling using a single-step kit [14, 15]. It features the THP ligand, which forms complexes with ^68^Ga rapidly at a low concentration at room temperature and over a wide pH range. Evaluations have demonstrated a favourable bio-distribution and affinity for targeting PSMA [14–16]. Whilst ^68^Ga-HBED-CC-PSMA PET-CT has been shown to impact management in between 39 and 62% of patients with BCR of prostate cancer after previous definitive treatment and in 21% of patients undergoing primary staging, no such data has yet been reported for ^68^Ga-THP-PSMA [17, 18].

We hypothesised that ^68^Ga-THP-PSMA PET-CT impacts clinical management in prostate cancer in high-risk (HR) patients before planned curative treatment and following BCR.

Our aim was to prospectively record changes in intended treatment plans in both groups of patients in a tertiary prostate cancer service.

## Materials and methods

### Patient data and management

A questionnaire was adapted from Roach et al. to record management plans before and after ^68^Ga-THP-PSMA PET-CT [17] (Appendix 1). Patients with high-risk (HR) prostate cancer (defined by the D’Amico classification) before surgery or radiation therapy, or patients with BCR, defined by the American Society for Therapeutic Radiology and Oncology (ASTRO) criteria, were eligible [19, 20]. This service evaluation was granted institutional approval by the Guy’s and St Thomas’ Service Evaluation committee and the requirement for consent for data use that was anonymised before analysis was waived.

A pre-PET-CT management plan was prospectively documented on the questionnaire at a multidisciplinary meeting (MDM) consisting of urologists, oncologists, histopathologists, radiologists and a nuclear medicine physician. PSA at diagnosis or at recurrence, PSA doubling time (PSAdt) in those with BCR and Gleason score at diagnosis or at recurrence were recorded. Results of conventional prior imaging were used to inform management. All patients undergoing primary staging have a multiparametric MRI as part of their routine diagnostic evaluation. Any subsequent validation information for the ^68^Ga-THP-PSMA PET-CT results, including histology and post-PSMA PET-CT conventional imaging up to 6 months, was evaluated.

The post-^68^Ga-THP-PSMA PET-CT management plan resulting from MDM discussion or clinical review was recorded on the questionnaire. Any cases where the management plan remained unclear were reviewed by a clinical oncologist who assessed the post-^68^Ga-THP-PSMA PET-CT scan treatment plan according to local and national treatment guidelines. The management change was recorded as either intra-modality or inter-modality. Inter-modality change was defined as an alteration in the type of management (e.g. cancellation of salvage radiotherapy (RT) due to poly-metastatic disease demonstrated on ^68^Ga-THP-PSMA PET-CT, whereas intra-modality change was defined as a modification of dose/site/strategy that was previously indicated.

### Radiochemistry

^68^Ga-THP-PSMA was prepared using a kit preparation (GalliProst™) containing 40.0 μg tris(hydroxypyridinone)-Glu-urea-Lys(Ahx), sodium bicarbonate, mannitol and phosphate buffer.

Gallium-68 from a Ge-68/Ga-68 generator with a Ge-68 breakthrough of ≤ 0.001 % was directly eluted into the kit vial. The generator was eluted within 5 mL of 0.1 M HCl(aq). The kit solution was incubated for 5 min at room temperature for complete labelling with a vent needle in situ to clear excess carbon dioxide produced during the labelling process.

^68^Ga-THP-PSMA was analysed according to the monographs 2462 (gallium chloride) and 2482 (gallium edotreotide) of the European Pharmacopeia. Radioanalytic thin layer chromatography (TLC) was performed using glass microfiber chromatography paper impregnated with a silica gel (Agilent technologies) and 10% ammonium acetate in water/methanol (030:70) and also with citrate cugger pH 5. The radio-iTLCs were analysed on a MiniGita thin-layer chromatography scanner.

High-performance liquid chromatography (radio-HPLC) was performed on a Varian ProStar high-pressure gradient system equipped with an ultraviolet-visible detector (Varian ProStar 335) and a radiodetector using a RP-18 column. Radiochemical purity and yield were > 95%.

### ^68^Ga-THP-PSMA PET-CT scan

No specific patient preparation was required except bladder voiding immediately before imaging. All patients were injected intravenously with ^68^Ga-THP-PSMA (mean 159 ± 21.2 MBq). At 60 min, a scan was acquired from pelvis to skull base at 4 min per bed position with an axial field of view of 15.7 cm and an 11-slice overlap between bed positions, using a GE Discovery 710 PET-CT scanner (GE Healthcare, Chicago, USA) at our institution. A low-dose CT scan (140 kV, mA 15–100, noise index 40, 0.5-s rotation time and 40 mm collimation) was performed at the start of imaging to provide attenuation correction and an anatomical reference. PET image reconstruction included scanner-based corrections for radiotracer decay, scatter, randoms and dead-time. Emission sinograms were reconstructed with an ordered subset expectation maximisation algorithm (2 iterations, 24 subsets) with the scatter correction performed using the absolute scaling of the scatter sinogram.

### Scan analysis

All scans were assessed by two PET specialists (GC/VW) in consensus using Hermes Hybrid Viewer (version 2.6, Hermes Medical Solutions, Stockholm, Sweden). To enable us to evaluate real life conditions, the reviewers had knowledge of the patients’ relevant clinical details including other imaging. A positive scan was recorded if prostate, prostate bed, lymph node, bone or focal visceral uptake was present above adjacent background tissues and could not be accounted for by physiological or normal variation uptake [14]. Consensus was reached on all scans between the two readers without the need to use a third reader. Scan results were made available to the referring clinician and prostate cancer MDM only after a pre-scan management plan had been documented.

### Statistical analysis

Data were tested for normality using the Shapiro-Wilk test. Normally distributed data were expressed as mean ± standard deviation. Non-normally distributed data were log-transformed before comparison or displayed as median with range. Statistical analyses were conducted using IBM SPSS statistics software (version 24). A *p* value of < 0.05 was used for statistical significance.

## Results

### Patient demographics

Fifty patients with high-risk, newly diagnosed prostate cancer and 68 patients with BCR were consecutively evaluated. Mean patient age was 65 years (range 48–85 years).

Demographic and pathological data are found in Table 1. Gleason score for the HR group is taken from prostate biopsy, whilst those from the BCR cohort are taken from biopsy and/or prostatectomy specimen as applicable. Ten cases were reviewed by a clinical oncologist, who provided clarification of the management plans.

**Table 1:** Patient and disease characteristics of 118 scanned patients

Characteristic	HR group	BCR group
Age in years	65 years (48–85)	68.2 years (49–85)
PSA level (μg/L)	38.72 (1.61–265.1)	4.44 (0.16–71.02)
Gleason score	7 (3 + 3 to 5 + 5)	7 (3 + 3 to 5 + 4)
6	2	5
7	32	42
8	5	6
9	9	15
10	2	0
Pathological stage		
T2a	7	5
T2b	9	20
T2c	6	10
T3a	10	16
T3b	18	15
T4	0	2
PSA doubling time	N/A	9.06 months (1–40.9)

Data are means with ranges in parentheses

### HR group

**Fig. 1:**
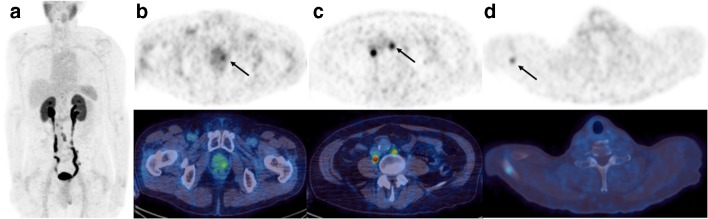
^68^Ga-THP-PSMA PET-CT scan of a man diagnosed with high-risk prostate cancer. **a** Maximum intensity projection image. **b** The primary tumour shows focal activity in the left peripheral zone (arrow). **c** There is evidence of retroperitoneal nodal disease (arrow) and **d** bone metastases (arrow). The detection of metastatic disease changed management from a surgical to systemic therapy approach with hormones and chemotherapy

The information acquired from ^68^Ga-THP-PSMA PET-CT in 12 of the 50 scans (24 %) led to a change in management. Nine were inter-modality and 3 were intra-modality (Table 2) (Fig. 1).

**Table 2:** Type of management change in primary diagnosis high-risk cohort following ^68^Ga-THP-PSMA PET-CT scan

Initial management plan	Revised management plan (*n* = 12)
RARP with PLND	RARP without PLND (*n* = 3)
RARP without PLND	RARP and PLND (*n* = 1)
	RT & ADT (*n* = 1)
	ADT alone (*n* = 1)
Radiotherapy (including in combination with ADT)	Brachytherapy (*n* = 1)
	ADT alone (*n* = 2)
	ADT and chemotherapy (*n* = 1)
ADT alone	RT and ADT (*n* = 1)
	ADT and chemotherapy (*n* = 1)

All ^68^Ga-THP-PSMA PET-CT scans demonstrated uptake within the prostate. The SUV_max_ was significantly higher (*p* < 0.0001) in prostate cancer lesions (mean 7.08; 95% CI 5.67–8.50) than in corresponding normal prostatic tissue (mean 1.89; 95% CI 1.68–2.09). The median ratio of SUV_max_ in prostate cancer lesions to normal tissue in the same patient was 3.1 (range 1.6–16.4).

All patients underwent a mpMRI scan as part of routine diagnostic evaluation prior to ^68^Ga-THP-PSMA PET-CT.

#### Correlation with clinical parameters

34.6% (9/26) of patients with a management change had a PSA < 20 μg/L whilst 12.5% (3/24) of patients with a management change had a PSA > 20 μg/L (Table 3).

**Table 3:** Management change (%) in primary diagnosis high-risk cohort as related to PSA and TNM stage

PSA	< 20 (*n* = 9/26) *34.6%*	> 20 (*n* = 3/24) 12.5%
N1	4	5
M1a	0	3
M1b	*4*	0

Gleason score did not affect the proportion of patients who had a management change (25.8% when Gleason < 8 and 25% if > 8) but Gleason score > 8 was associated with more N1 disease (25%, 4/16 vs 16.1%, 5/31) and more bone metastases (12.5%, 2/16 vs 6.5%, 2/31) (Table 4).

**Table 4:** Management change (%) in primary diagnosis high-risk cohort as related to Gleason score and TNM stage

Gleason	< 8 (*n* = 8/31) 25.8%	> 8 (*n* = 4/16) 25%
N1	16.1%	*25%*
M1a	6.5%	6.3%
M1b	6.5%	15

#### Staging

^68^Ga-THP-PSMA PET-CT identified 42 patients with N0 disease and 8 with N1 disease compared with 41 N0 and 9 N1 on mpMRI. Regarding the presence of distant metastases, 3 extra-pelvic lymph nodes (M1a) and 4 bone (M1b) lesions were identified on ^68^Ga-THP-PSMA PET-CT compared with 2 M1a and 2 M1b on mpMRI and bone scan.

#### Treatment plan

In 8 patients, pelvic lymph node dissection (PLND) was planned at the time of radical prostatectomy, as they were identified as ‘high-risk’ as per the D’Amico classification. If pathological lymph nodes are identified on imaging, as per standard UK practice, radical surgery is generally not offered to patients, and radiotherapy or systemic treatments are recommended instead. Following the ^68^Ga-THP-PSMA PET-CT scan, pathological lymph nodes were identified in 2/8 patients, with the remaining 6 having negative scans for nodal disease. One patient underwent a radical prostatectomy with PLND (as per patient choice) with histologically positive nodes and the second underwent radical radiotherapy and hormone treatment. Of the remaining 6 patients (with negative ^68^Ga-THP-PSMA PET-CT imaging), 4 underwent a PLND due to high-risk clinical features. All removed lymph nodes were benign. Two patients did not undergo PNLD and are currently free of recurrence.

#### Follow-up

Follow-up is available for a median of 17 months (range 1–23 months). In 46 patients, the ^68^Ga-THP-PSMA PET-CT correctly staged the patient, as reflected by their current follow-up, with the majority of patients on PSA surveillance following radical treatment (robot-assisted radical prostatectomy (RARP) or RT with an undetectable PSA (defined as < 0.03 μg/L). One patient has been referred back to his original hospital and we do not have further follow-up data.

Three patients are having additional treatment. One had RARP without PLND (no nodes identified by ^68^Ga-THP-PSMA PET-CT). The PSA remained elevated post-operatively and the patient has commenced hormones and radiotherapy to the prostate bed on the assumption that residual disease was present. The second patient had a positive node on pre-RARP ^68^Ga-THP-PSMA PET-CT. PLND was conducted but post-operative PSA remained elevated and repeat ^68^Ga-THP-PSMA PET-CT confirmed further nodal disease. The patient is now on hormone and radiotherapy treatment. The final patient had a positive surgical margin and therefore required radiotherapy to the prostatic bed.

### BCR group

**Fig. 2:**
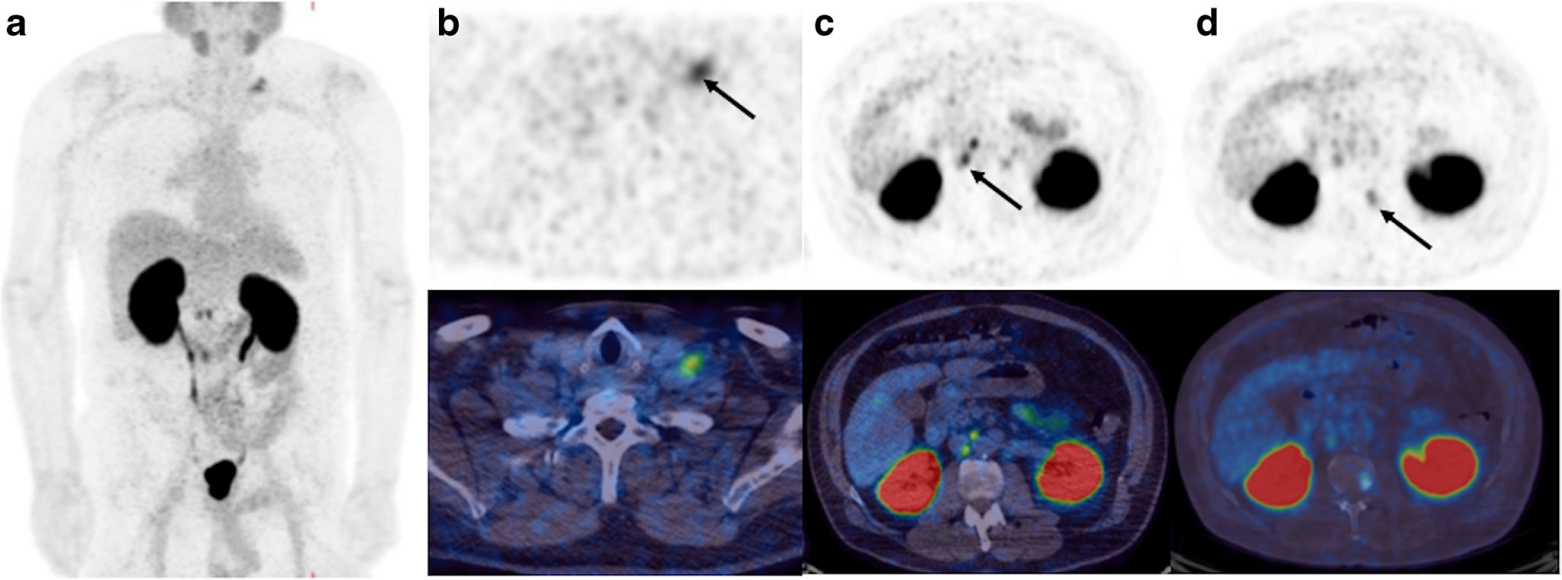
^68^Ga-THP-PSMA PET-CT scan of a man with a rising PSA (3 μg/L) after a previous radical prostatectomy. **a** Maximum intensity projection image. **b** There is evidence of nodal disease in the left supraclavicular fossa (arrow) and **c** retroperitoneum (arrow) as well as **d** a vertebral bone metastasis (arrow)

Twenty-three of 68 (34%) patients had ^68^Ga-THP-PSMA PET-CT scans that resulted in a management change. Seventeen of the 23 (74%) were inter- rather than intra-modality management changes, and details are outlined in Table 5 (Fig. 2). Forty of the 68 ^68^Ga-THP-PSMA PET-CT scans demonstrated positive uptake (59%). The mean time from primary treatment to BCR was 28 months (range 4–62 months) and 8 patients had a recurrence within 9 months of their initial treatment.

**Table 5:** Type of management change in BCR cohort following ^68^Ga-THP-PSMA PET-CT

Management plan pre-PSMA PET-CT	Patients who had management changed	New management plan post-PSMA PET-CT	Patients who did not have management changed
Surveillance	3	ADT (*n* = 1)	11
		ADT and chemotherapy (*n* = 1)	
		SABR (*n* = 1)	
RARP	2	Surveillance (*n* = 1)	6
		ADT (*n* = 1)	
Brachytherapy	2	Surveillance (*n* = 1)	1
		Watchful waiting (*n* = 1)	
SABR	2	EBRT (*n* = 1)	5
		ADT (*n* = 1)	
EBRT	5	Surveillance (*n* = 1)	13
		ADT (*n* = 2)	
		SABR (*n* = 1)	
		RARP (*n* = 1)	
ADT	9	Best supportive care (*n* = 1)	9
		ADT and chemotherapy (*n* = 3)	
		Chemotherapy (*n* = 3)	
		EBRT (*n* = 1)	
		SABR (*n* = 1)	

*ADT* androgen deprivation therapy, *SABR* stereotactic ablative radiotherapy, *EBRT* external beam radiotherapy, *RARP* robotic-assisted radical prostatectomy

#### Correlation with clinical parameters

**Fig. 3:**
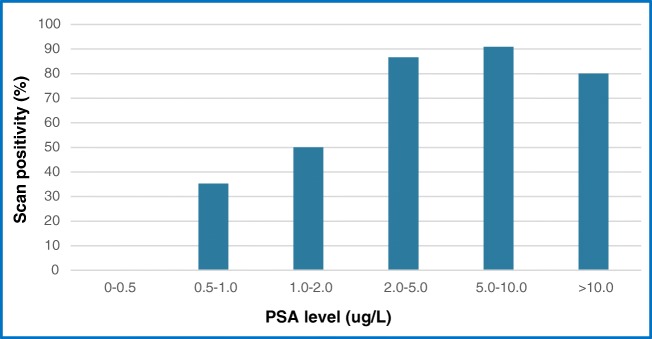
Scan positivity in BCR at different PSA levels

**Fig. 4:**
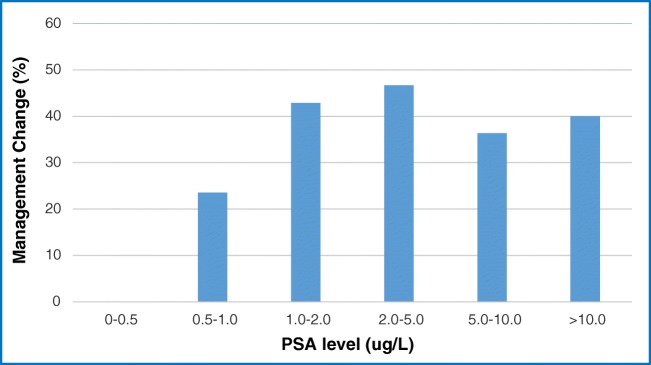
Management change as related to PSA value in BCR group

^68^Ga-THP-PSMA PET-CT scan positivity was compared with PSA values. Fifty nine percent of scans were positive (PSA range 0.82–71.0 μg/L (mean 6.34)) whilst 41% were negative (PSA range 0.11–14.2 μg/L mean 1.7, *p* < 0.001) (Fig. 3). No positive scans were identified when the PSA was < 0.5 μg/L. Management change was seen in 4/17 (23.5%) patients with PSA levels between 0.5 and 1.0 μg/L with higher but broadly similar proportions of changes in management in patients with PSA 1.0–2.0 μg/L (6/14, 42.9%), PSA 2.0–5.0 μg/L (7/15, 46.7%), PSA 5.0–10.0 μg/L (4/11, 36.4%) and PSA > 10.0 μg/L (2/5 (40%) (Fig. 4).

**Fig. 5:**
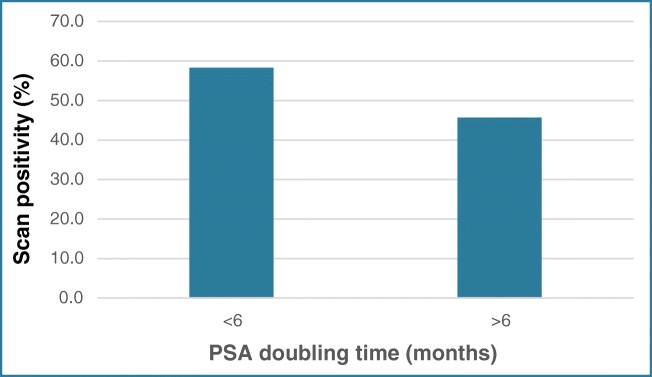
Scan positivity as related to PSA doubling time in BCR group

PSAdt at diagnosis was available for 60 patients and Gleason scores for 62, as a proportion of patients were diagnosed and treated in external hospitals. For those with a PSAdt < 6 months, 14/25 (56%) had a positive scan compared with 16/35 (45.7%) > 6 months (Fig. 5). Management changed was in 9/25 (36%) of those with PSAdt < 6 months and 9/35 (25.7%) of those with PSAdt > 6 months.

**Fig. 6:**
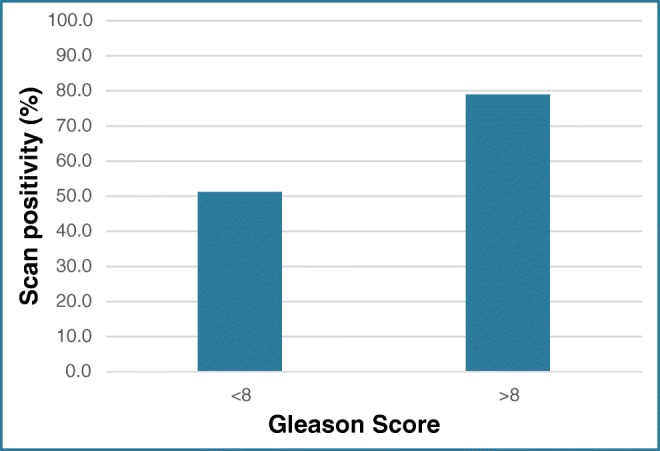
Scan positivity as related to Gleason score in BCR group

Twenty-two out of 43 (51.2%) patients with a Gleason score < 8 had a positive scan compared with 15/19 (78.9%) of those with a Gleason score > 8 (Fig. 6). Management changed was in 13/43 (30.2%) patients with Gleason < 8 and in 7/19 (36.8%) of patients with Gleason > 8.

#### Follow-up

Follow-up is available for a median of 13 months (range 2 to 18 months) for the total cohort from the date of the ^68^Ga-THP-PSMA PET-CT. Currently, 27 men are on PSA surveillance, 22 on androgen deprivation therapy (ADT), 10 are having chemotherapy (2 with hormone-sensitive disease, 8 with castrate-resistant metastatic prostate cancer), 4 undergoing radiotherapy, 2 on watchful waiting, 2 having best supportive care (due to metastatic tumours of non-prostatic origin) and 1 being considered for salvage surgery. In 61 of the 68 BCR patients, subsequent follow-up has not been discordant with ^68^Ga-THP-PSMA PET-CT results.

In 7 patients, the ^68^Ga-THP-PSMA PET-CT scan was negative, but all patients have now been taken off PSA surveillance. In 5 of these patients, despite a negative ^68^Ga-THP-PSMA PET-CT scan, there is ongoing concern about micro-metastatic disease due to a continuing rise in PSA; 1 is having more intensive PSA surveillance (PSA 1.42 μg/L), 2 have commenced ADT (PSA 1.98 μg/L and 1.2 μg/L, respectively) and 2 are on chemotherapy (PSA 1.37 μg/L and 1.67 μg/L, respectively). In the other 2 patients, the initial ^68^Ga-THP-PSMA PET-CT scan was reassuring. However, due to rising PSA, the scan was repeated (at 6 and 9 months, respectively), and nodal disease has now been demonstrated. The first of these two patients is on ADT and the other on watchful waiting.

Whilst the majority of patients had multi-modal treatment and subsequently underwent a ^68^Ga-THP-PSMA PET-CT scan, only minor differences were identified between those with previous primary RARP compared with RT. 34.7% of patients who had a RARP had a change in management compared with 39.1% who had RT.

## Discussion

Our study has demonstrated a clinically significant impact on the management of patients with high-risk prostate cancer and BCR, imaged using ^68^Ga-THP-PSMA PET-CT.

### Primary staging in high-risk prostate cancer

In our series of patients undergoing ^68^Ga-THP-PSMA PET-CT for primary staging, a management change (compared with the decision made in an MDM using standard diagnostic techniques) was noted in 12 of the 50 patients (24%). Roach et al. evaluated a comparable cohort of 108 patients scanned for primary staging and reported a 21% management change [17].

We noted a higher number of management changes in patients with PSA < 20 μg/L (34.6%) compared with patients with PSA > 20 μg/L (12.5%). This implies that at high PSA levels, conventional staging with MRI, bone scan and/or CT can detect nodal or metastatic disease, but at lower PSA levels, ^68^Ga-THP-PSMA PET-CT has greater sensitivity and management impact. We did not find a difference in management changes between patients with Gleason score < 8 and > 8.

Perera et al. analysed 16 studies involving 1309 patients who underwent a ^68^Ga-PSMA PET-CT (most used ^68^Ga-HBED-CC-PSMA, some did not mention the specific ligand), of which 40% of scans were positive for patients undergoing primary staging (95% CI 19–64%) [12]. Four studies were evaluated for the predictive ability of PSMA PET-CT (scan positivity compared with histology), demonstrating a sensitivity of 80% and specificity of 97%.

Lymph node evaluation is integral to primary staging and there is a need for imaging which provides more sensitive detection in high-risk prostate cancer. Current practice regarding lymphadenectomy is reliant on pre-operative models using PSA levels, Gleason score and T-stage to dictate surgical planning [19].

Whilst our aim was not to specifically measure the accuracy of nodal detection, ^68^Ga-THP-PSMA PET-CT identified lymph nodes in 8/50 patients (16%). In 2 patients, identification of lymphadenopathy led to a management change: one to RARP with a unilateral PLND (histologically positive node) and one to radiotherapy and hormones instead of RARP.

A number of studies have evaluated the sensitivity and specificity of ^68^Ga-HBED-CC-PSMA PET-CT for lymph node detection. Sensitivity is reported as between 33.3–65.9% and specificity 98.9–100% [21, 22]. Budäus et al. reported that ^68^Ga-PSMA PET-CT scans identified 4 of 12 patients (33.3%) as node positive. In 8 patients with histologically confirmed lymph nodes, ^68^Ga-PSMA PET-CT was negative, giving a false-negative value of 66.7%.

Current EAU guidelines recognise that PLND are associated with worse peri- and postoperative outcomes, whilst a direct therapeutic effect is still not evident from the current literature. Thus, it is not our routine practice to offer surgery and lymph node dissection to patients with pathological nodes on imaging. A recent review of UK practice in 2017 demonstrated that most radical prostatectomies are performed for patients with Gleason grade group 2 and 3 disease (intermediate risk). Lymph node dissection rates were similar across all grade groups ranging from 13–15% [23].

In our cohort, ^68^Ga-THP-PSMA PET-CT detected 7 patients with metastatic disease at primary presentation (3 M1a and 4 M1b), which was greater than that identified by conventional imaging alone. Treatment choice did change as a result of these findings in 4/7 patients (57%).

### Biochemical recurrence

In our BCR group undergoing ^68^Ga-THP-PSMA PET-CT, clinical management changed in 23 of the 68 patients (34%), with 59% of scans demonstrating positivity. In 23 scans, disease recurrence was identified locally (prostate or prostate bed), 23 in lymph nodes and in 9, metastatic disease was seen. In those where there was a management change, 10 patients were upstaged (42%) and 2 downstaged (8.3%), and in 12 scans (50%), staging was concordant with that of conventional imaging, but additional information relevant to treatment planning was identified.

Roach et al. in Australia and Afaq et al. in the UK have shown 62% and 39% change in management intent, respectively, in the BCR setting, with the use of ^68^Ga-HBED-CC-PSMA [17, 18]. Roach et al. have conducted the largest prospective multicentre study of 323 patients evaluating management intent. They evaluated all patients undergoing imaging for BCR, with a detectable PSA but negative conventional imaging. Overall clinical intent changed in 51% of patients following results of PSMA PET-CT. ^68^Ga-HBED-CC-PSMA PET-CT detected additional local disease in 27% of patients, nodes in 39% and metastatic disease in 16%.

The systematic review by Han et al., evaluating management changes following ^68^Ga-HBED-CC-PSMA PET, was performed for BCR in 11 studies [24]. The proportion undergoing RT increased from 56 to 61%, typically with an increase in dose or target volume. There was an increase in patients undergoing salvage surgery from 1 to 7%, which included performing pelvic lymph node dissection (in our group, this decreased from 10.2 to 5.9%). The number of patients undergoing systemic treatment decreased from 26 to 12% (in our cohort, it increased from 5.9 to 10.2%). ADT was initially planned in 144 patients but was only commenced in 52 patients after ^68^Ga-HBED-CC-PSMA (in our group, planned in 28% and decreased to 25%). The proportion of patients with no treatment decision or on surveillance decreased from 14 to 11% (we report an increase from 29.4 to 38%). In addition, we report a small decrease in numbers undergoing radiotherapy (from 26.4 to 21%).

At PSA levels of 1.0 μg/L and above, we noted higher rates of management change (0% < 0.5 μg/L, 23% 0.5–1 μg/L, 46% 1–2 μg/L). Higher scan positivity and change in management rates were also seen in patients with short PSAdt < 6 months (56% vs 45.7% and 36.0% vs 25.7%, respectively). Similarly, those with higher Gleason scores > 8 had higher scan positivity rates (78.9% vs 51.2%) but broadly similar changes in management (36.8% vs 30.2%).

Fendler et al. using ^68^Ga-HBED-CC-PSMA PET-CT reported a detection rate of 75% in BCR. There was a significant increase in detection rate across PSA ranges, 38% for < 0.5 ng/mL, 57% for 0.5–< 1 ng/mL and 84% for 1.0 ng/mL [25]. There were 8 cases where the PET findings were reported as negative but histology confirmed prostate cancer.

Similar trends were identified in a retrospective series by Ceci et al. regarding factors which correlated with ^68^Ga-HBED-CC-PSMA PET-CT detection in patients presenting with BCR [26].

^68^Ga-HBED-CC-PSMA PET-CT was positive in 23 of 39 patients (59 %) with PSA < 2 μg/L. These results are consistent with data recently reported by Afshar-Oromieh et al. who reported scan positivity rates with ^68^Ga-HBED-CC-PSMA PET-CT of 61.1% in 90 patients with PSA < 2 μg/L [27]. PSA kinetics were evaluated, reporting that ^68^Ga-HBED-CC-PSMA PET-CT was positive in 85% of patients with low PSA values and short PSAdt, but in only 18.7% of patients with low PSA and long PSAdt. For Perera et al. in the BCR cohort, ^68^Ga-HBED-CC-PSMA PET positivity increased as serum PSA rose. For those with a PSA < 0.2 μg/L, the detection rate was 42%, which increased to 58%, 76%, and 95% for the 0.2–0.99, 1.00–1.99, and > 2.0ug/L PSA values, respectively [12]. A similar trend was demonstrated with PSAdt, with a PSMA positivity of 92% for those with a PSAdt < 6 months and 64% for those ≥ 6 months. The authors again recognise the significant heterogeneity between the groups analysed.

Several studies have determined that the clinical detection rate was dependent on the PSA level, with Derlin et al. reporting rates as high as 94.1% for PSA > 10 μg/L, 54.5% for PSA 1–2 μg/L and 20% below 0.5 μg/L [28]. Afaq et al., using ^68^Ga-HBED-CC-PSMA, reported an overall scan positive rate of 15.8% (PSA 0.2–0.5 μg/L), 33% (PSA 0.5–1 μg/L), 20% (PSA 1–2 μg/L) and 84.6% (PSA 2–5 μg/L) [18].

The difference in management change and scan positivity rates between these studies and ours might be explained by differences in prospective versus retrospective methodology, referral patterns and decision-making between Australian, European and UK practice. The data from Afaq et al., a UK study in the same city, is more comparable with ours and hence the closer values for management impact [18].

Whilst previous studies have reported higher detection rates with other PSMA ligands, ^68^Ga-THP-PSMA demonstrated reasonable detection rates in patients with BCR and PSA levels ≥ 1 μg/L. Potential explanations for the lower detection rates at very low PSA levels using this ligand compared with ^68^Ga-HBED-CC-PSMA include different use of ADT between studies which alters PSMA expression, faster renal clearance of THP compared with HBED and potential lower affinity of ^68^Ga-THP-PSMA. No comparisons have been performed to determine if this impacts on diagnostic accuracy or management.

### ^68^Ga-THP-PSMA

^68^Ga-THP-PSMA can be labelled to Good Manufacturing Practice criteria in 5 min at room temperature as a single-step technique and has a potential practical and cost advantage over other ^68^Ga-labelled PSMA radiopharmaceuticals. Hofman et al., Young et al. and Derlin et al. have evaluated this ligand for its safety and bio-distribution, whole-body radiation dose, plasma clearance and correlation of uptake with tumour PSMA expression on histopathology [14, 15]. They demonstrated that ^68^Ga-THP-PSMA had lower background uptake in salivary glands, liver and spleen than ^68^Ga-HBED-CC-PSMA. Metastatic lesions were equally identified on both HBED-PSMA and THP-PSMA-PET scans. ^68^Ga-THP-PSMA provides a similar effective dose to that published for ^68^Ga-HBED-CC-PSMA (2.07 × 10^−2^ mSv/MBq).

### Limitations

This is a single-centre study and may not reflect practice in other units where diagnostic and treatment guidelines may differ. Nevertheless, the results reflect practice in a busy tertiary referral centre with a large surgical and oncological prostate cancer service. Management decisions were made in a multidisciplinary fashion for a consecutive cohort of patients. The study reports intended management and did not take into account actual treatment, which may on occasion differ from planned due to patient comorbidities or other patient-related factors. Not all of the PET-positive finding scans were validated by another procedure such as histological analysis of lymph nodes or visceral lesions (not always feasible) or conventional imaging.

## Conclusions

Our study of a new radiopharmaceutical PSMA formulation that can be rapidly manufactured from a ^68^Ga generator and cold-kit vial demonstrates that ^68^Ga-THP-PSMA PET-CT impacts on management decisions in HR prostate cancer prior to radical therapy and BCR, despite lower scan positivity rates at PSA levels < 0.5 μg/L. Scan positivity is related to PSA level, PSA doubling time and Gleason score.
